# The SOS Pilot Study: A RCT of Routine Oxygen Supplementation Early after Acute Stroke—Effect on Recovery of Neurological Function at One Week

**DOI:** 10.1371/journal.pone.0019113

**Published:** 2011-05-19

**Authors:** Christine Roffe, Khalid Ali, Anushka Warusevitane, Sheila Sills, Sarah Pountain, Martin Allen, John Hodsoll, Frank Lally, Peter Jones, Peter Crome

**Affiliations:** 1 Stoke Stroke Research Group, North Staffordshire Combined Healthcare Trust, Stoke-On-Trent, Staffordshire, United Kingdom; 2 Institute for Science and Technology in Medicine, Keele University, Keele, Staffordshire, United Kingdom; 3 Academic Departments of Geriatrics, Brighton and Sussex Medical School, Brighton, East Sussex, United Kingdom; 4 Department of Respiratory Medicine, University Hospital of North Staffordshire, Stoke-On-Trent, Staffordshire, United Kingdom; 5 School of Computing and Mathematics, Keele University, Keele, Staffordshire, United Kingdom; 6 Institute for Life Course Studies, Keele University, Keele, Staffordshire, United Kingdom; Julius-Maximilians-Universität Würzburg, Germany

## Abstract

Mild hypoxia is common after stroke and associated with poor long-term outcome. Oxygen supplementation could prevent hypoxia and improve recovery. A previous study of routine oxygen supplementation showed no significant benefit at 7 and 12 months. This pilot study reports the effects of routine oxygen supplementation for 72 hours on oxygen saturation and neurological outcomes at 1 week after a stroke.

**Methods:**

Patients with a clinical diagnosis of acute stroke were recruited within 24 h of hospital admission between October 2004 and April 2008. Participants were randomized to oxygen via nasal cannulae (72 h) or control (room air, oxygen given only if clinically indicated). Clinical outcomes were assessed by research team members at 1 week. Baseline data for oxygen (n = 148) and control (n = 141) did not differ between groups.

**Results:**

The median (interquartile range) National Institutes of Health Stroke Scale (NIHSS) score for the groups at baseline was 6 (7) and 5 (7) respectively. The median Nocturnal Oxygen Saturation during treatment was 1.4% (0.3) higher in the oxygen than in the control group (p<0.001) during the intervention. At 1 week, the median NIHSS score had reduced by 2 (3) in the oxygen and by 1 (2) in the control group. 31% of participants in the oxygen group and 14% in the control group had an improvement of ≥4 NIHSS points at 1 week doubling the odds of improvement in the oxygen group (OR: 2.9).

**Conclusion:**

Our data show that routine oxygen supplementation started within 24 hours of hospital admission with acute stroke led to a small, but statistically significant, improvement in neurological recovery at 1 week. However, the difference in NIHSS improvement may be due to baseline imbalance in stroke severity between the two groups and needs to be confirmed in a larger study and linked to longer-term clinical outcome.

**Trial Registration:**

Controlled-Trials.com ISRCTN12362720; European Clinical Trials Database 2004-001866-41

## Introduction

Hypoxia is common after acute stroke, affecting up to 63% of patients at some time after admission [1]–[4]. Hypoxia may have significant adverse effects on the ischaemic brain after stroke. While healthy adults with normal cerebral circulation can compensate for mild hypoxia by an increase in cerebral blood flow [5], this is not possible in the already ischaemic brain after stroke [6]–[8]. There is a clear association between hypoxia, neurological deterioration and mortality after stroke [4], [9], [10]. Specialist care on stroke units is effective in preventing death and disability [11]. Patients on stroke units are less likely to have hypoxic events [12] and more likely to receive oxygen than patients on a non-specialized general ward [13].

Oxygen treatment is not without side effects [14]. It impedes early mobilization, dries mucous membranes, could lead to upper and lower airway infection, and may disrupt sleep [15]. There is evidence from animal models and *in vitro* studies that oxygen encourages the formation of toxic free radicals leading to further damage to the ischaemic brain [16]–[19], especially during reperfusion [20]. Oxidative stress has also been implicated in the activation of cell signalling pathways which lead to apoptosis and neuronal cell death [21], [22] Other experimental studies, however, suggest that eubaric hyperoxia reduces free radical generation in the ischaemic and reperfused brain [23]–[25].

There is only one large (n = 550) quasi-randomized study of oxygen supplementation for acute stroke, and this suggests that routine oxygen treatment to unselected stroke patients does not reduce morbidity and mortality [26]. A more recent, very small (n = 16), study of short term high flow oxygen treatment (45 L/min for 8 hours) after acute stroke showed transient early improvements in neurological performance and infarct size but no long-term clinical benefit at 3 months [27]. A meta-analysis of clinical trials of routine oxygen supplementation for acute myocardial infarction showed no benefit, and potential harm [28]. In a previous study we have shown that low flow (2 L/min) oxygen supplementation for 24 hours is well tolerated, raising oxygen saturation by 2.5% and increasing the proportion of patients with an oxygen saturation >90% throughout the night from 23% to 59% [29].

The evidence for oxygen treatment after acute stroke from experimental and clinical studies is conflicting, and, unsurprisingly, stroke management guidelines differ while based on the same sparse evidence [30]–[32]. It is therefore important to provide better evidence to support clinical decision making.

In this paper we report on a pilot study examining the effects of routine fixed dose oxygen supplementation at a rate of 2 or 3 L/min dependent on baseline oxygen saturation for 72 hours on oxygen saturation and neurological recovery at 1 week.

## Methods

The protocol for this trial and supporting CONSORT checklist are available as supporting information; see Checklist S1 and Protocol S1.

### Trial design, setting, and subjects

This is a randomized controlled single blind pilot study of routine oxygen supplementation after acute stroke. A double blind study could not be used since medical staff would know the group allocated due to the presence/absence of nasal cannulae. Patients were recruited from the University Hospital of North Staffordshire (UHNS), UK, a large teaching hospital admitting about 800 patients with stroke per year. Depending on bed availability, most stroke patients are admitted to the acute stroke unit within 24 hours of presentation. Patients with an admission diagnosis of stroke or possible stroke were identified by a member of the stroke research group, who checked the medical admissions unit log book every morning and was contactable by the medical admissions team for new strokes in the day. Adult patients with a clinical diagnosis of acute stroke [33] were eligible for inclusion if they were admitted to UHNS within the preceding 24 hours, able to give informed consent, or a relative was contactable and willing to give assent, and if there was no clear indication for or against oxygen treatment. The final decision as to whether the patient had a definite clinical need for or contraindication against oxygen treatment was left to the clinician treating the patient. Reasons for not including patients in the trial were:

- Recognised indications for oxygen treatment such as oxygen saturation on air <90%, acute left ventricular failure, severe pneumonia, pulmonary emboli, and chronic respiratory failure treated with long term oxygen at home.
- Recognised contraindications to fixed dose oxygen treatment (2–3 L/min via nasal cannulae), e.g. type II respiratory failure.
- Stroke was not the patient's primary clinical diagnosis. Patients with other serious life-threatening illnesses likely to lead to death within the next few months were also excluded.

### Intervention

Participants were randomized to one of two treatment groups, the oxygen group and the control group. Participants in the oxygen group were given oxygen at a flow rate of 2 L/min if baseline oxygen saturation (SpO_2_) was greater than 93% or at a rate of 3 L/min if baseline SpO_2_ was 93% or less. This dose was based on the results of earlier studies [29], [34] and aimed at keeping the oxygen saturation within the normal range. Oxygen was administered via nasal cannulae continuously for 72 hours from the time of recruitment. Short discontinuation of oxygen treatment was permitted in mobile patients during trips to the toilet and to physiotherapy, the time periods for these were not recorded but understood to be no more than one hour. Participants in the control group were not given routine oxygen supplementation. Heart rate, blood pressure, and SpO_2_ were assessed regularly (at least three times a day) as part of routine clinical care. Those who developed indications for oxygen, or needed a higher concentration of oxygen than the protocol prescribed, were given the concentration of oxygen they needed by the treating clinician, irrespective of the treatment group they were in.

### Randomization

Due to a protocol change after the first year, two randomisation methods were used. Randomization for the first 153 participants into either arm of the study, was via telephone, the researcher rang the randomization number and declared the intent to randomize. The operator first allocated the patient the next number in a computer-generated sequence, then recorded basic clinical data and on completion disclosed the treatment allocation to the researcher who would initiate the treatment. Once this sequence was complete, the patient was included in the trial whether treatment was subsequently administered or not.

From the second year, 148 participants were randomized, into either arm, via a computer-based portal operating in a similar sequence to the telephone system described above. Due to randomness of the allocations between the systems there was a small imbalance in numbers to each of the 2 treatment arms; a total of 155 for oxygen and 146 to control. However, we do not believe that this has led to the introduction of systematic errors.

### Baseline clinical assessment and follow-up

At baseline, details of the medical history were established by interview and consultation of medical notes. Patients were examined neurologically and classified as total anterior circulation syndrome (TAC), partial anterior circulation syndrome (PAC), lacunar syndrome (LAC) and posterior circulation syndrome (POC) using the Oxfordshire Community Stroke Project (OCSP) Classification [35]. Neurological deficit was scored using the National Institute for Health Stroke Scale (NIHSS) [36] and the Scandinavian Stroke Scale (SSS) [37]. The NIHSS was included because it is the most widely used stroke scale and allows comparisons with other ongoing studies, the SSS was also included to allow comparison with the oxygen supplementation study by Ronning and Guldvog [26] For both scales death was recorded as the worst possible score on the scale (35 for NIHSS and 0 for SSS). Aetiology was determined by computed tomography of the head and reported as cerebral infarct or intracerebral haemorrhage. Haemorrhagic infarcts and scans with non-specific findings compatible with but not diagnostic of acute cerebral infarction were recorded as infarcts. Both infarcts and haemorrhages were included because waiting to confirm the diagnosis of ischaemic stoke before recruitment would have delayed treatment unnecessarily and there is no good reason to suspect that oxygen would harm patients with intracerebral haemorrhage, or that it is more effective in that group. Pulse oximetry was performed on the second night of the intervention. At 1 week the NIHSS was repeated and indicators for potential adverse effects of oxygen treatment such as stress (tachycardia and hypertension), behaviour disturbance (sedative use), and infection (temperature and antibiotic use) were recorded.

### Pulse oximetry

Oxygen saturation and heart rate were assessed using a Pulsox-3I pulse oximeter (Konica Minolta). The instrument records SpO_2_ every second producing a moving average every 5 seconds using an internal algorithm. Hands were inspected to ensure that the fingers were warm and well-perfused. Nail varnish was removed and long fingernails were clipped, where necessary. The pulse oximeter was attached to the wrist and the sensory probe was fitted to the index finger. To reduce movement artifacts the hemiparetic side was used for oximetry [38]. Pulse oximetry was performed overnight from 21:00 to 09:00 on the second night after recruitment. On completion the recorded, data were downloaded onto a Personal Computer (PC) using Oximeter Download Software (Stowood Scientific Instruments, Oxford, UK). The first 5 min of the recording were defined as the baseline awake oxygen saturation and oxygen desaturation was defined as a 4% fall in saturation compared to baseline. Readings between 23:00 and 07:00 were taken as nocturnal oxygen saturation. If oximetry was unsuccessful or incomplete (less than 4 hours) it was repeated on night 3. Recordings with data for less than 4 h were not included in the final analysis. Recordings which were shorter than 8 h but at least 4 h long results for T<90 (the time subjects spent with a SpO_2_<90%) was corrected to a notional 8 h period using the formula: T<90 (in minutes)  =  (T<90 in minutes/actual recording time in minutes) x 480. The same corrections were used for T<80 and T>98.

### Ethical approval, trial registration, and consent

The protocol was approved by the North Staffordshire Research Ethics Committee on 06.10.2004 (ref: 04/Q2604/73). It is registered in the International Standard Randomized Controlled Trial Number Register (ISRCTN12362720) and the European Clinical Trials Database (EudraCT number 2004-001866-41). Written informed consent was sought from all study participants. Assent from the next of kin was accepted if the patient agreed to take part but was unable to give fully informed consent. Participants who were incompetent at the time of recruitment, but competent when followed-up at 1 week were asked to confirm consent. Patients who were recruited to the study by assent and refused consent at 1 week were withdrawn from the study.

### Statistical Analysis

Descriptive statistics and counts were performed using Microsoft Excel, Microsoft Office XP, Microsoft Corporation, US, and tests of significance (as detailed in the text) were carried out using SPSS statistical software version 15.0 (SPSS Inc., Chicago, Illinois, US). Statistical significance was accepted if the p-value was <0.05.

## Results

### Recruitment

**Figure 1:**
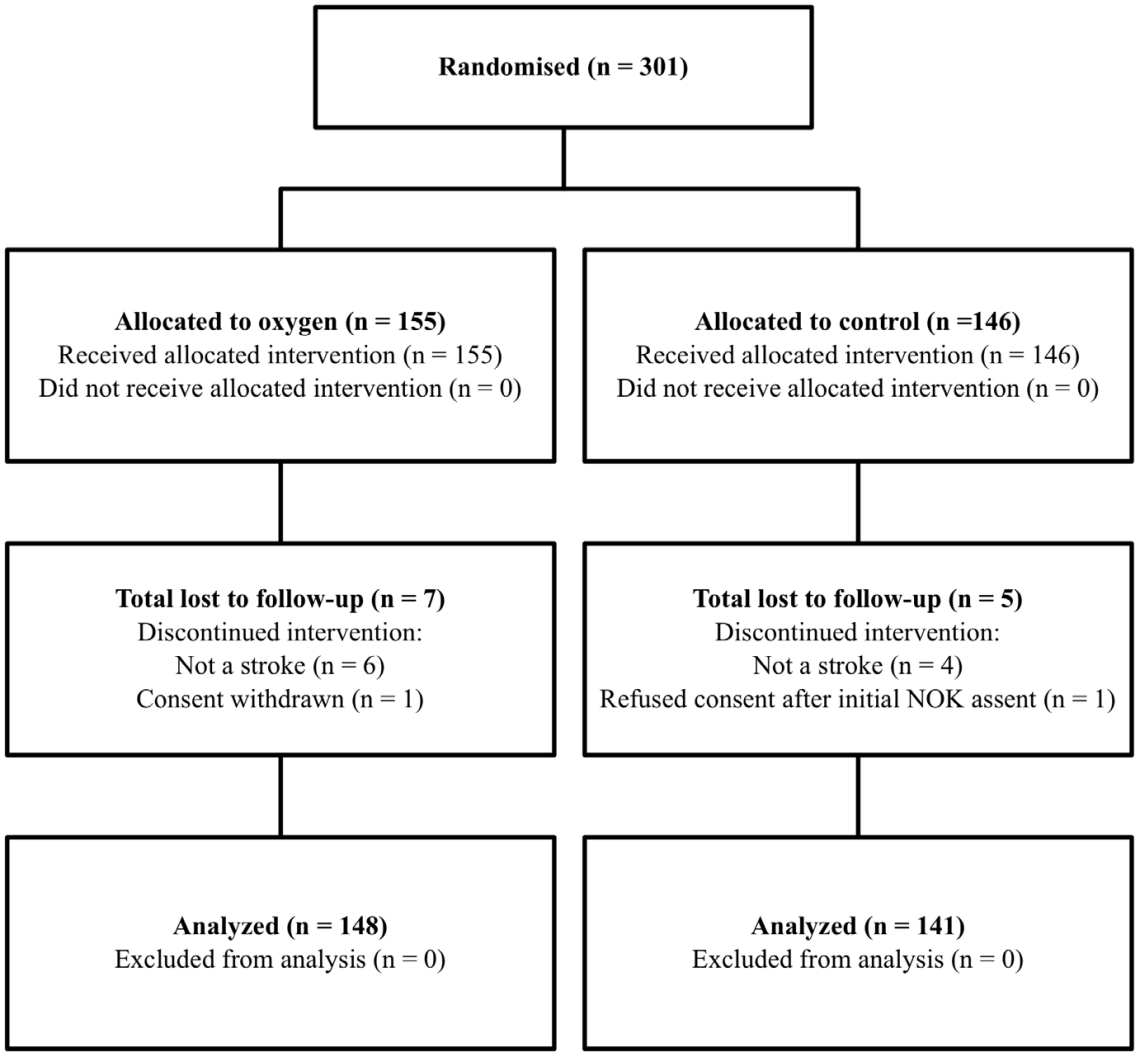
Consort diagram illustrating the flow of participants through the study. NOK: Next of Kin.

Three hundred and one patients were recruited to the study from October 2004 to April 2008, with a mean recruitment rate of 7 (range 1 to 23) per month; the flow of patients through each stage of the study is shown in figure 1. The initial clinical diagnosis of stroke was shown to be incorrect in 6 participants in the oxygen group and 4 in the control group. After further investigation these were diagnosed as: brain tumour n = 7, motor neurone disease n = 1, possible multiple sclerosis n = 1 and no final diagnosis n = 1. At one week follow-up neurological data were available for 143 (97%) and 133 (92%)in the oxygen and control group respectively. Oximetry data were available for 98 (66%) participants in the oxygen group and 100 (71%) participants in the control group. Reasons for missing data are shown in figure 1.

### Baseline demographic and clinical data

Baseline demographic and clinical characteristics, stroke type and severity, and oxygen saturation at randomization were similar in the control and oxygen group (table 1).

**Table 1:** Baseline characteristics of the study population.

	Oxygen(n = 148)	Control(n = 141)
**Demographic characteristics**		
Mean Age (years)	73 SD 11.7	71 SD 11.5
Male sex (n,)	65 (44%)	72 (51%)
**Prognostic factors**		
Living alone (n)	61 (41%)	52 (37%)
Independent in basic activities of daily living (n)	122 (82%)	121 (86%)
Normal verbal response (n)	102 (69%)	90 (67%)
Able to lift affected arm (n)	92 (62%)	91 (65%)
Able to walk (n)	29 (14%)	20 (15%)
**Concomitant medical problems**		
Ischaemic heart disease (n)	34 (23%)	37 (26%)
Congestive cardiac failure (n)	16 (11%)	18 (13%)
Atrial fibrillation (n)	34 (23%)	19 (14%)
Chronic obstructive pulmonary disease/asthma (n)	14 (10%)	12 (9%)
**Details of the stroke**		
Mean Time since stroke hh∶mm)	17∶48 SD 8∶5	16∶31 SD 8∶2
Stroke pathology (n% cerebral infarct)	134 (91)	119 (85)
Glasgow Coma Scale Score (median, IQR)	15 (0)	15 (0)
Scandinavian Stroke Scale Score (median, IQR)	39 (15)	42 (18)
National Institute for Health Stroke Scale (median, range)	6 (7)	5 (7)
Total anterior circulation syndrome (n)	24 (17%)	25 (18%)
Partial anterior circulation syndrome (n)	47 (34%)	46 (34%)
Lacunar syndrome (n,)	58 (42%)	57 (42%)
Posterior circulation syndrome (n)	6 (4%)	6 (4%)
Transient ischaemic attacks or unclassified (n)	4 (3%)	3 (2%)
**Other Clinical data**		
Oxygen saturation at randomization (mean)	96.1% SD 1.9	96.1% SD 2.0

SD = Standard deviation

The mean age of patients included in the study was 72.3 (SD 11.6) years, 137 (47%) were male, 243 (84%) were independent for activities of daily living before the stroke, and 113 (39%) were living alone. Concomitant medical problems included ischaemic heart disease in 71 (25%), congestive cardiac failure in 34 (12%), atrial fibrillation in 53 (18%), and chronic obstructive pulmonary disease in 26 (9%) of participants. The majority of patients were normoxic with a mean oxygen saturation of 96.1% (SD 1.9%, range 90–100%) at randomization. The mean time from stroke onset to randomization was 17∶12 (SD 8∶42) hh∶min. Bamford classification data is included in table 1. The final diagnosis at 1 week was cerebral infarct in 254 (88%), intracerebral haemorrhage in 23 (8%), subdural haemorrhage in 1 (0.3%), transient ischaemic attack in 6 (2%), and undetermined (no CT head scan) in 7 (2%). The median Glasgow Coma Scale score (IQR) was 15 (0) and 15 (0) for the control and oxygen groups respectively. The level of neurological deficit was assessed by the NIHSS scale; median scores were 6 (7) and 5 (7) for the oxygen and control group respectively.

Inclusion was via fully informed consent in 115 (78%) participants in the oxygen group and 106 (75%) in the control group and by assent in 33 (22%) participants in the oxygen group and 35 (25%) in the control group. At the 1 week reassessment, patients who were included via assent were asked to confirm consent if they had regained competence. One patient in the control group and 1 patient in the oxygen group refused consent at that point and were withdrawn.

### Oxygen saturation

Oxygen saturation was assessed 3 times a day during the first 72 hours as part of routine clinical practice. In 48 (32%) patients in the oxygen group and 52 (47%) patients in the control group one or more readings showed an SpO_2_ of less than 90% (p = 0.4, Chi squared test). Twelve patients (8%) in the oxygen group and 16 patients in the control group (11%) were prescribed oxygen for clinical indications during the treatment period (p = 0.4 Fisher's Exact test).

Pulse oximetry was conducted successfully in 98 patients in the oxygen group and in 100 controls. All parameters of oxygenation were significantly better in the oxygen than in the control group except the time spent with an SpO_2_ of less than 80% and 90% and the number of participants who spent more than 1 hour with a saturation of less than 90%. The median Mean Nocturnal Oxygen Saturation was 1.3% higher in the treatment group than in controls (p<0.001, Mann-Whitney U test). A lower proportion of patients in the oxygen group spent more than 5 min and more than 30 min with an SpO_2_ of <90% during the recording night (p<0.05, Chi squared test). There was no significant difference in the proportion of patients who spent more than 60 min with an SpO_2_ of less then 90%, or in the time spent with an SpO_2_ of less than 80%, since such very severe hypoxia was uncommon, and would have been treated as soon as identified by the clinical team.

Eighty-nine participants (29%) had no or insufficient oximetry data for analysis. This equated to missing values of 41 and 48 for the controls and oxygen group respectively. There was no significant (p = 0.5, Fischer's Exact test) difference in the proportion of patients returning no or insufficient oximety data between the two groups. Participants who did not complete oximetry were older than those who completed (74.1 vs. 71.4 years), less likely to have been independent before the stroke (73% vs. 89%), less likely to have full scores on the GCS verbal response item (57% vs. 71%), less likely to be able to walk (8% vs. 17%), and were recruited sooner after stroke onset (15∶20 vs. 17∶10 hh∶mm). Otherwise there were no significant differences in baseline. Further information on oxygen saturation is provided in table 2.

**Table 2:** The effect of routine oxygen supplementation on oxygen saturation.

	OxygenGroup(n = 98)	ControlGroup(n = 100)	p-value
Awake Oxygen Saturation (median, IQR, %)	97.0 (1)	95.9 (1.9)	<0.001^a^
Mean Nocturnal SpO_2_ (median, IQR, %)	96.0 (2.7)	94.7 (2.0)	<0.001^a^
Difference between SpO_2_ at randomization and awake SpO_2_ (median, IQR, %)	0.4 (2.8)	−0.4 (3.1)	<0.001^a^
Lowest Nocturnal SpO_2_ (median, IQR, %)	91.0 (6.0)	89.0 (6.0)	0.007^a^
ODI 4% (median, IQR)	0.3 (1.4)	0.9 (3.2)	0.005^a^
Tc<90% (median, IQR, hh∶mm)	0 (0∶04)	0∶02 (0∶13)	NS^a^
Tc<80% (median, IQR, mm∶ss)	0∶0 (0∶0)	0∶0 (0∶0)	NS^a^
Tc>98% (median, IQR, hh∶mm)	1∶31 (4∶55)	0∶02 (0∶20)	<0.001^a^
More than 5 min Tc<90% (n,%)	20 (20%)	33 (33%)	<0.05^b^
More than 30 min Tc<90% (n,%)	8 (8%)	17 (17%)	<0.05^b^
More than 60 min Tc<90% (n,%)	4 (4%)	5 (5%)	NS^b^

a Mann Whitney U-test; ^b^Chi-squared test; NS: Not significant; ODI 4%: 4% Oxygen Desaturation Index

### Neurological outcome at 1 week

Analysis was by intention to treat. Neurological scores improved from baseline to 1 week in both groups. At baseline the median (IQR) NIHSS was 6 (7) in the oxygen and 5 (7) in the control group. By 1 week the median NIHSS scores (including deaths) had improved more in the oxygen than in the control group −2 (3) for oxygen vs. −1 (2) for control, p<0.001 Mann Whitney U-test). A significant improvement in neurological scores was defined as reduction of 4 or more points in the NIHSS from baseline to week 1. The odds ratio (95% CI) for significant improvement with oxygen supplementation was 2.9 (1.59–5.4). Forty five (31%) participants showed a significant improvement in neurological score in the oxygen group compared to 18 (14%) participants in the control group (table 3). There was no difference in the number of deaths in both groups (4 in the control and 5 in the oxygen group, p = 1.0 Fisher's Exact Test).

**Table 3:** Neurological recovery at one week.

	OxygenN = 148	ControlsN = 141	P value
**NIHSS at randomisation**			
Median (min; max)	6 (0; 29)	5 (0; 31)	NS
IQR	7	7	
**NIHSS at week 1**			
Median (min; max)	2.5 (0; 35)	3 (0; 35)	NS
IQR	6*	7**	
**NIHSS difference between baseline and week 1**			
Median (min; max)	−2 (−13; 29)	−1 (−26; 27)	<0.001^a^
IQR	3	2	
**Patients with an NIHSS improvement** ≥**4 at week 1**			
n (%)	45 (31)*	18 (14)**	<0.0001^b^

There were no data for the one week NIHSS in 5 patients in the oxygen group and 8 patients in the control group thus n = 143* for oxygen and n = 133** for control for the analysis of difference between baseline and week 1 NIHSS. NS: not significant; ^a^Mann Whitney U-test; ^b^Chi-squared test.

### Exploratory subgroup analysis

**Figure 2:**
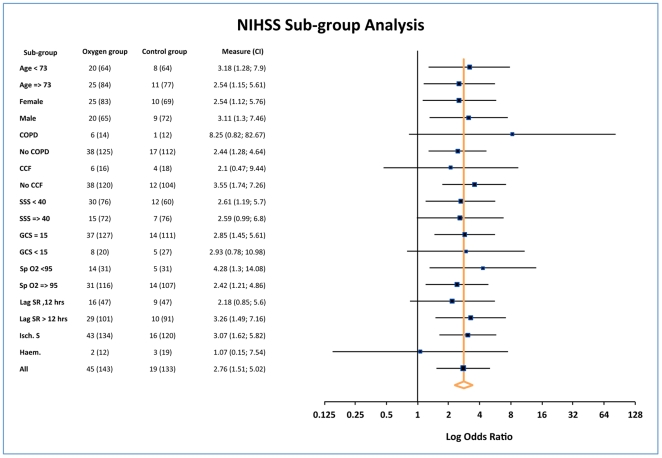
Forest plot of the odds of a 4 point or greater improvement of the National Institute for Health Stroke Scale (NIHSS) score between randomization and week 1. The figures in the oxygen and control columns are the number of events (NIHSS improvement) followed by the total number in brackets which comprises events and non-events. The figure shows that the odds of improving with oxygen treatment were similar in all the subgroups tested. The apparent adverse effect in the haematoma group is not significant, and likely to be due to very small numbers of patients with improvement at one week in this subgroup (one improved in the oxygen group versus 2 on the control group. COPD: chronic obstructive pulmonary disease, CCF: congestive cardiac failure, SSS: Scandinavian Stroke Scale, GCS: Glasgow Coma Scale, SpO2: oxygen saturation, Lag SR: time lag between stroke onset to recruitment.

Oxygen treatment might be more effective in older patients with a history of heart and lung problems, those with lower baseline oxygen saturation, patients with more severe strokes, or those with an impaired level of consciousness. It might also be affected by treatment with oxygen before randomization and the aetiology of the stroke (infarct or haemorrhage). We therefore carried out an exploratory subgroup analysis to identify subgroups which might benefit more or less from oxygen treatment. The odds ratios for significant improvement with oxygen treatment (NIHSS reduction from baseline of 4 or greater) [39] for each of these subgroups are shown in figure 2. The study was not powered for subgroup analysis, but the results suggest that there was no significant difference in the effect for any of the subgroups. The odds ratio for NIHSS improvement in intracerebral haemorrhages was below 1 (OR = 0.69), but the number of participants in this group was too small to be reliable and hence the confidence interval was very wide.

### Other outcomes at 1 week

There was no significant difference in the group means for oxygen and control respectively for highest systolic blood pressure (167 SD 29 mmHg vs. 167 SD 28 mmHg), highest diastolic blood pressure (93 SD 19 mmHg vs. 91 SD 15 mmHg), and heart rate (92 SD 16 BPM vs. 92 SD 19 BPM) during the 72 hours of the treatment period. The highest mean temperature during the first week was similar in both groups (37.2% SD 0.6 vs. 37.1% SD 0.7 C). There was no difference in the proportion of patients requiring antibiotics in the first week (n = 27 (18%) vs. n = 22 (15%) for oxygen and control respectively) or sedative medications (n = 9 (6%) vs. n = 9 (6%) for oxygen and control respectively).

## Discussion

The main finding of this study is that routine oxygen supplementation, given for 72 hours at a rate of 2 or 3 L/min, dependent on baseline oxygen saturation leads, to a small but statistically significant improvement of neurological recovery at 1 week. The odds of improving by 4 or more NIHSS points at 1 week were doubled in the oxygen group. While statically significant the difference in the improvement in NIHSS is small and may be due to the non-significant baseline imbalance in stroke severity between the two groups (baseline NIHSS was 1 point higher in the oxygen than in the control group). The NIHSS is not designed as an outcome tool, and the relevance of a small change in NIHSS during the first 7 days on longer-term functional outcome is unclear.

However, other studies have shown that baseline NIHSS is a strong predictor of recovery and long-term outcome [40]–[42]. Reanalysis of data from the TOAST (Trial of Org10172 in Acute Stroke Treatment) Study showed that a 1-point change in NIHSS from baseline to 3 months was associated with a doubling of the odds of a very favourable outcome (Glasgow Outcome Scale Score of 1 and modified Barthel index>18) at 3 months [43].

Our data contrasts with an earlier study by Ronning and Guldvog [26] which showed no benefit from routine oxygen supplementation. This is likely not due to differences in patient population; those of Ronning and Guldvog are comparable to our study population (mean age 76, median SSS 42/43 and the proportion of infarcts 87%/87% in strokes and controls in the Ronning study). The difference in outcome may be due to: Different time points of assessment (7 months and 1 year for Ronning and Guldvog, 1 week in this study), longer duration of treatment (72 hours in this study, 24 hours in the other) Keep the same order of comparison e.g Ronning V SOS, a different dose of oxygen (3 L/min for Ronning vs. 2 L/min in 93% and 3 L/min in 7% of the oxygen group in this study), or due to poor compliance with the prescribed treatment (11% in the oxygen group were not given oxygen for 24 hours and 26% of controls were prescribed oxygen for clinical indications in the Ronning and Guldvog study). Additionally, the Ronning and Guldvog study did not report oxygen saturation before and during treatment. It is therefore impossible to say whether the observed lack of effect may have been due to failure to improve oxygen saturation on treatment, or due to oxygen toxicity and free radical formation in patients with an oxygen saturation at the higher end of the normal scale. Subgroup analyses in the Ronning and Guldvog [26] study suggested that patients with milder strokes may have been harmed by oxygen. The results of our study do not confirm this, but, conversely, suggest that both mild and more severe strokes benefited similarly from oxygen supplementation. Neither our study nor that of Ronning and Guldvog [26] was sufficiently powered to support subgroup analysis and the dangers of false positives are acknowledged [44] therefore differences could have been due to chance. Nevertheless, the dose of oxygen given (2 L/min in the majority) and the duration of treatment were different in our study than in the Ronning and Guldvog study, and this could explain the difference in outcomes.

A more recent, but small, study by Singhal *et al* [27] (n = 16) supports our findings. They dosed oxygen not just to keep oxygenation within normal range, but to act pharmacologically as a therapeutic agent. This was achieved by increasing the arterial oxygenation above normal (oxygen at a rate of 45 L/min given over 8 hours). NIHSS scores in the treated group were higher at 4h, 24 h, 1 week and 3 months, but this trend only achieved statistical significance at 24 h. Magnetic resonance imaging at the same time points showed improved penumbral salvage in the oxygen group, which achieved statistical significance at 4 h only. A larger study is ongoing. Even more intensive oxygen treatment using hyperbaric oxygenation has been tested in acute stroke patients. A meta-analysis of 3 randomized controlled trials of hyperbaric oxygen (n = 106) concluded that the sample was too small to provide clear guidelines for practice, but that a significant clinical effect was unlikely [45].

While we aimed to include a representative sample of all stroke patients admitted to the hospital, the majority of subjects included had relatively mild strokes. The median NIHSS at baseline in this study was 6 and 5 in the oxygen and control groups respectively, while the median NIHSS of patients admitted to the local stroke unit is about 8 (unpublished data from clinical audit 2008/2009). The main reason for this is the requirement of informed consent or assent and the short time between admission and recruitment. While patients with milder strokes can give informed consent, those with more severe strokes can only be included if assent from the next of kin can be gained. By the time subjects are identified for the study, relatives have usually left hospital and are then contacted during visiting times or have to travel back in to discuss the study. Patients with more severe strokes were more likely to benefit from oxygen in the Ronning and Guldvog study [26] and under representation of this group in our study is likely to reduce the level of benefit observed. More importantly, it leaves doubts about the transferability of the results to more severe strokes, and may mean that those who are most likely to benefit may not be given the treatment because of lack of evidence. It is important to include a wide range of patients in acute stroke studies, and independent physician consent should be considered in this situation. Independent physician consent for acute stroke studies is supported by user group consultations [46], [47]. Ten patients included in the study had to be excluded later because they did not have a stroke. Most of the excluded patients had brain tumours. Patients were included on the basis of the clinical presentation, and the final diagnosis was made at 1 week when the result of the head scan was available. Since the number of exclusions was similar in both groups, this should not have affected the results.

The results of the overnight pulse oximetry showed that oxygen supplementation effectively improved all parameters of oxygenation. Mean Nocturnal Oxygen Saturation was 1.3% higher in the oxygen group than in the control group. In two smaller preparatory studies we have shown that oxygen supplementation at a rate of 2 L/min during the day with compliance ensured by continuous observation raises oxygen saturation by 2% [34] and that the same dose given overnight and observed intermittently (2 am and 3 am) increases nocturnal oxygen saturation during the first night after the start of treatment by 2.5%. [29] The differences between these and the current study are likely to be due to compliance. Smaller studies allow closer observation and thus better compliance with treatment. Moreover, oxygen saturation here was assessed on night 2 rather than immediately after the start of treatment in the previous studies and this again, may have allowed lower compliance rates. The results of the present study, which required no additional observation over and above the usual routine in acute stroke units, most closely resembles current clinical practice, and will therefore be directly applicable to usual clinical care. The need to document and monitor compliance with oxygen prescription more closely has recently been stressed in the British Thoracic Society Guidelines for emergency oxygen use, where it is suggested correct administration of oxygen should be checked and signed off by the nurse at each drug round (e.g. five times a day) [48]. It is of note that implementation of this guidance on stroke units is likely to lead to better compliance and may improve outcomes further.

Oxygen supplementation is simple, cheap, and applicable even in the most basic clinical environments. The results of this study suggest that oxygen given at 2 L/min for 72 h may improve neurological recovery at 1 week. The results of this pilot study should be confirmed in a larger fully powered trial, which is now ongoing (The Stroke Oxygen Study) [49]. The findings have led to a number of modifications of the Stroke Oxygen Study protocol. Independent physician consent was included to allow more patients with severe stroke to be included in the trial. Additional data collection on oxygen saturation and treatment adherence was included to document compliance with the allocated trial treatment. The difference in NIHSS between recruitment and week one was included as a pre-specified secondary outcome.

## Supporting Information

Checklist S1 CONSORT Checklist.
(DOC)

Protocol S1 Trial Protocol.
(DOC)
