# Supplementary material for: The SOS Pilot Study: A RCT of Routine Oxygen Supplementation Early after Acute Stroke—Effect on Recovery of Neurological Function at One Week
Source: PLoS One. 2011 May 19;6(5):e19113. doi: 10.1371/journal.pone.0019113 (PMC3098237; doi:10.1371/journal.pone.0019113)
Supplement: Protocol S1 — Trial Protocol. (DOC) [file pone.0019113.s002.doc]

**S**troke **O**xygen **S**tudy

A randomised controlled **pilot study** of the effects of routine oxygen supplementation on functional outcome after acute stroke

Version 2 (24.06.2004)

**Investigators team:**

C. Roffe (main investigator)

K. Ali (main researcher)

P. Jones (statistics)

M. Allen (respiratory medicine advisor)

R K Hills (clinical trial unit support)

P. Crome

**WHAT IS THE PRINCIPAL RESEARCH QUESTION/OBJECTIVE?**

**Main Hypothesis**

The main hypothesis to be tested is whether routine fixed dose oxygen treatment during the first 3 days after an acute stroke will reduce death and disability during the first 6 months after the stroke.

Stroke patients will only be included in to the study if the indication for oxygen is uncertain. Patients with definite indications or contraindications to oxygen will be treated according to established clinical practice.

It is possible that the proposed pilot study is too small to answer the research question, but it is necessary to inform the ensuing larger multi-centre study.

**The main objectives for the pilot trial are:**

To assess differences in death and disability (Rankin, Nottingham EADL and EuroQuol)at 6 months between the oxygen treatment and the control groups. This will be used to calculate the sample size for the planned multi-centre trial.

To assess potential side effects of oxygen treatment. These may include confusion and restlessness because of poor sleep, delayed return to mobility because of the physical restraint of the oxygen tubing and infections arising from oxygen insufflation.

To assess the frequency of protocol violation (oxygen given outside the trial protocol). This will further inform the sample size calculation for the multi-centre study.

To assess the feasibility of recruiting stroke patients within 24 hours of hospital admission. The recruitment rate will inform the number of centres required for a future multi-centre study of oxygen treatment.

**SCIENTIFIC BACKGROUND**

**1. Why is there a need to investigate the effects of oxygen supplementation after stroke?**

It is now well-established that specialist care on stroke units is effective in preventing death and disability after stroke.[[1]](#endnote-2) It does, however, still remain unclear which aspects of stroke care are crucial for improving outcome. It has been shown that patients on a stroke unit are more likely to receive oxygen than on a non-specialised general ward.[[2]](#endnote-3) Oxygen treatment may thus be a factor relevant to stroke outcome. Mild hypoxia is common in stroke patients and may have significant adverse effects on the ischaemic brain after stroke.[[3]](#endnote-4) While healthy adults with normal cerebral circulation can compensate for mild hypoxia by an increase in cerebral blood flow,[[4]](#endnote-5) this is not possible in the already ischaemic brain after stroke.[[5]](#endnote-6) [[6]](#endnote-7) [[7]](#endnote-8) For all these reasons the use of oxygen treatment is rapidly increasing in European stroke units. A questionnaire survey of UK stroke physicians showed that almost 50% of respondents would start oxygen supplementation after stroke at an oxygen saturation of 95% or above,[[8]](#endnote-9) which is well within the normal physiological range.[[9]](#endnote-10)

There are, however, also reasons for caution. Oxygen treatment is not without side effects.[[10]](#endnote-11) It impedes early mobilisation, poses an infection risk, and may encourage the formation of toxic free radicals leading to further damage to the ischaemic brain.[[11]](#endnote-12) Although in a non-randomised study, the work of Ronning et al have recently suggested that routine oxygen treatment to unselected stroke patients does not reduce morbidity and mortality. Subgroup analyses have further suggested that patients with severe strokes were more likely to benefit than those with mild strokes, but the study size was not sufficient to define patients who are likely to derive benefit.[[12]](#endnote-13) Routine oxygen supplementation for acute myocardial infarction has been abandoned after a clinical trials showed no benefit, and potential harm.[[13]](#endnote-14)

Other than the (non-randomised) study by Ronning suggesting that routine oxygen supplementation of all stroke patients may not be effective there is no evidence from controlled trials to guide oxygen therapy in acute stroke. In particular, there is no genuinely randomised evidence, and therefore no unbiased estimate, of the effectiveness of oxygen supplementation. Recommendations from different Stroke Managemtn guidelines are vague and conflicting.[[14]](#endnote-15) [[15]](#endnote-16) [[16]](#endnote-17) There is great uncertainty amongst physicians treating patients with stroke about which treatment approach to take, and when to give oxygen, as shown by a recent survey of British Stroke Physicians.[[17]](#endnote-18)

For all the above reasons it is important to identify groups of patients who benefit from oxygen, and those who do not.

**What is already known about oxygen supplementation after acute stroke?**

Ronning and Guldvog have suggested that giving oxygen at a rate of 3l/min to all stroke patients during the first 24 hours after hospital admission may not improve overall outcome. However, it is unclear whether this result suggests evidence of an absence of benefit or absence of evidence of benefit. Further, they have not reported baseline oxygen saturation, or changes in saturation on treatment. It is therefore possible that some patients were undertreated, and others achieved too high oxygen levels leading to an increase in free radical generation in the ischaemic penumbra.[[18]](#endnote-19) There are no other data from clinical studies to inform recommendations for the dose of oxygen to give. The recently updated European Stroke Initiative suggests a dose of 2-4 litres/minute,[[19]](#endnote-20) the American Stroke Association Guideline recommends keeping the oxygen saturation at or above 95%,[[20]](#endnote-21) but neither is based on evidence from randomised controlled trials. In the absence of data to the contrary it is reasonable to assume that treatment should aim to restore oxygen saturation to the normal range.

Normal oxygen saturation for adults is 95-98.5%,[[21]](#endnote-22) although in healthy older individuals it is lower at 95% ±2.5%.[[22]](#endnote-23) In a study of the prevalence of hypoxia in stroke patients conducted in North Staffordshire we have shown that oxygen saturation in stroke patients who are normoxic at recruitment is about 1% lower than that of age-matched community controls.[[23]](#endnote-24) We have just completed a dose titration study for oxygen after acute stroke and found that 2l/min oxygen by nasal cannula increases oxygen saturation by 2% and 3 l/min by 3%.[[24]](#endnote-25) [[25]](#endnote-26)We also found that oxygen masks were less likely to be tolerated than nasal cannulae, leading to poorer treatment compliance with the former. For the proposed study we will therefore give oxygen by nasal cannula. A dosage regime of 3l/min for individuals with a baseline oxygen saturation ≤93% and 2l/min for individuals with a baseline saturation >93% is likely to prevent hypoxia without increasing oxygen saturation beyond the upper limit of the normal range, and this regime will therefore be used in the proposed study.

There is also no consensus on for how long to continue oxygen supplementation.[[26]](#endnote-27) Data from a local study suggest that the changes in oxygen saturation during the first few days after the stroke cannot be predicted reliably. Over a 3-day period about a third deteriorate, a third remain stable and a third improve.[[27]](#endnote-28) Cells in the ischaemic penumbra may be viable for up to 16 hours in humans,[[28]](#endnote-29) and in baboons an increase in the size of the ischaemic penumbra has been shown between 24 hours and 17 days after the induced stroke, suggesting brain tissue may be at risk of deteriorating long after the ischaemic event. In the only clinical trial of oxygen supplementation after acute stroke published so far oxygen was given for 24 hours, and did not improve outcome.[[29]](#endnote-30) It is possible that a longer duration of treatment may be more effective. For this pilot study a treatment duration of 72 hours has been chosen. This will allow treatment to cover the time when the ischaemic penumbra may still be viable, and the vulnerable period early after the stroke.

# STUDY DESIGN

A randomised controlled open pilot study of routine fixed dose oxygen supplementation versus no routine oxygen treatment.

**DETAILED PLAN OF INVESTIGATION**

**Summary of the study**

This is a study of the effect of routine oxygen supplementation early after acute stroke. A brief synopsis of the trial structure is given in the attached Flow Chart. Subjects will be randomised to receive fixed dose oxygen or no routine oxygen for 72 hours. Oxygen saturation will be recorded over night by pulse oximetry on night 2. The patient will be assessed by a member of the research team on day 3 and week 1 after trial enrolment. At 3 months the researcher will consult the hospital computer system and the patient's notes to find out whether the patient was discharged from hospital and to where. At 6 months after trial enrolment the patient will be sent a questionnaire with questions relating to their daily functional abilities.

**Recruitment**

Patients will be recruited from the University Hospital of North Staffordshire by a member of the research team or the doctor admitting the patient. Because hypoxia is more likely to be detrimental early after the stroke, when the ischaemic penumbra either recovers or expands it is crucial to include patients as soon as possible after the stroke into the trial. Patients will therefore be recruited as soon as they enter hospital via MAU or A&E, if possible, or at the next earliest opportunity up to 24 hours after admission.

# Eligibility for trial inclusion

There are no definite guidelines for oxygen treatment after acute stroke, and there is uncertainty amongst stroke physicians about who should be given oxygen treatment. The eligibility criteria reflect this uncertainty, and allow for randomisation of all acute stroke patients who do not have definite indications or definite contraindications for oxygen treatment.

Adult patients will be eligible for trial inclusion if

- They were admitted with symptoms of an acute stroke within the preceding 24 h
- In the doctor's opinion there is no clear indication for and no clear contraindication against oxygen treatment.
- The patient is able to give consent , or a relative can be contacted to seek assent.

Reasons for not entering patients may include*:*

1. *Recognised indications for oxygen treatment such as* :

- Oxygen saturation on air ≤90%
- Hypoxia associated with acute left ventricular failure, severe pneumonia and pulmonary embolus
- Chronic respiratory failure treated with long term oxygen at home

1. *Recognised contraindications to fixed dose oxygen treatment (2-3l/min/vy nasal tubes)*

- Type II respiratory failure (contraindication to fixed dose oxygen as given in this trial)

3. *The stroke is not the patient's main clinical problem*

- The patient has another serious life-threatening illness likely to lead to death within the next few months

The final decision for trial eligibility rests with the responsible doctor assessing the patient.

**Diagnosis of stroke**

The diagnosis of stroke will be made by history and clinical examination and is at the discretion of the doctor admitting the patient. It will be based on the WHO criteria (rapidly developing clinical signs of focal (or global) disturbance of cerebral function, with symptoms lasting 24 h or longer, or leading to death, with no apparent cause other than of vascular origin).[[30]](#endnote-31) Within the first 24 hours of symptom onset a definite distinction between a stroke and a transient ischaemic attack cannot be made. However, most patients who still have persistent symptoms after one hour will be confirmed to have a stroke. Since waiting for 24 hours for confirmation would unnecessarily delay treatment we omitted the time element from the definition of stroke for the purposes of trial inclusion

**The uncertainty principle**

Further inclusion and exclusion criteria are not specified precisely, but are guided by the uncertainty principle. If the clinician is convinced (for whatever reason) that the patient should be treated, that patient should not be randomised, but should be given open label oxygen. If the clinician is convinced that (for whatever reason) the patient should not be given oxygen, the patient should not be included in the trial. Only patients who fulfil the inclusion criteria and for whom the clinician is uncertain about the benefit of oxygen treatment should be randomised. The uncertainty principle allows for inclusion of a wide range of patients and the detailed data collection and analysis will enable us to determine and describe the patient group who most benefits from oxygen (in the planned larger study). The uncertainty principle has been successfully used in large trials (e.g. MRC European Carotid Surgery Trial)[[31]](#endnote-32) [[32]](#endnote-33) and is still being used in ongoing stroke trials (e.g. the IST-3 study of thrombolysis for acute stroke currently in progress in our hospital and many others nationally and internationally).[[33]](#endnote-34)

**Intervention**

Patients will be randomised to one of two treatment groups:

1. Treatment group 1: no routine oxygen supplementation during the first 3 days of randomisation.
2. Treatment group 2: oxygen per nasal cannula continuously (day and night) at a flow rate of 3l/min if baseline oxygen saturation is ≤93% or at a rate of 2l/min (if baseline oxygen saturation  >93%) with humidification during the first 3 days after randomisation. Patients who develop definite indications or contraindications for oxygen treatment during the trial will be treated according to clinical need, irrespective of study treatment allocation.

**Blinding**

This study will be open, since placebo treatment (room air via nasal cannulae) would have at least some of the side effects of active treatment (e.g. infection and immobilisation) without any potential benefit, and would thus bias the data in favour of the treatment group. The main outcomes will be ascertained at 6 months by postal questionnaire. It is likely that participants will have forgotten which treatment group they were in 6 months after the intervention, and it is therefore unlikely that treatment allocation will bias their answers. We will assess whether patients remember their treatment allocation by including this as a question in the 6 month assessment questionnaire.

**Consent**

Fully informed consent will be sought from all competent subjects. In subjects who are conscious, but not fully competent to comprehend the information given and make a reasoned decision we will provide a simple explanation of the trial and seek the patient's agreement, but also seek assent from the next of kin. In subjects unable to participate in the decision process in any way we will seek assent of the next of kin only. If an incompetent subject has been included in the trial without giving fully informed consent we will strive to obtain fully informed consent as soon as the patient is able to do so. Consent will be sought by the doctor randomising the patient or by a member of the research team. All doctors randomising patients into the study will be given appropriate information about the trial treatment, potential effects and side effects and the assessment protocol by a member of the research team.

*The reason for including patients who are unable to give fully informed consent*

It is crucially important to include as wide a spectrum of stroke subjects as possible, and, in particular, patients with severe strokes. Patients with severe strokes may be more likely to develop hypoxia, and a recent study has suggested that patients with severe strokes may be more likely to benefit from oxygen than patients with mild strokes. However, patients with severe strokes are more likely to be confused, drowsy or dysphasic and thus unable to give informed consent. Exclusion of subjects unable to give informed consent is thus likely to bias trial outcome.

*The reason for involving the doctor admitting the patient in gaining consent for the study*

There are two major reasons for involving the admitting doctor in the consent process. First, it is likely that any potential neuroprotective effect of oxygen is most pronounced early after the stroke when cells in the ischaemic penumbra are still viable. It is thus important to include patients as soon as possible after admission into the trial to increase the chance of the treatment being beneficial. Second, many emergency units currently prescribe oxygen to stroke patients without good evidence from controlled trials to support this. In the design of this study it is important to match the trial protocol as closely as possible to the therapeutic situation it is designed to assess. While a member of the trial team can be available over the telephone for advice on a 24 hour daily basis, it would be impossible for the researchers to see all potential patients personally on admission. Oxygen is given routinely to patients in the medical emergency department and the medical admissions unit. All doctors know the effects and contraindications, and are thus competent to prescribe oxygen. The research team will make sure that recruiting doctors are aware of the protocol and principles of obtaining consent for research studies.

**Subject/ Patient participation**

This trial does not impose any major risks or burdens upon the patient.

The patient will be given supplemental oxygen via nasal tubes for 3 days or no routine oxygen depending on treatment group. Any patient who develops a definite indication or contraindication for oxygen during the trial will be treated as clinically indicated. Oxygen is widely used and safe in the patient group eligible for study inclusion. We do not expect any major adverse events. Potential minor adverse effects of oxygen are discomfort produced by the nasal cannulae, drying of mucous membranes, and limitation of mobility. On night 2 of the study oxygen saturation will be monitored over night with a pulse oximeter attached to one hand. This may affect the patient's mobility. In most patients the oximeter will be attached to the paralysed hand, and will therefore not interfere with movement. The researcher will ask the patient a few questions on day 3 and examine the patient after one week. This should take a maximum of 5 minutes and does not involve removal of clothes. At 3 months the researcher will consult the hospital records to find out to where the patient has been discharged. At 6 months the patient will be sent a questionnaire asking questions about how the stroke has affected their ability to get on with their daily activities. This may take up to 30 minutes to complete. If the researcher cannot contact the patient by mail or by phone at 6 months they might contact the GP or district nurses to find out how he/she is doing.

**Randomisation and initial assessment**

This assessment is limited to the minimum baseline clinical data required for randomisation and clinical assessment (See Randomisation Form) and incorporates validated tools (Glasgow Coma Scale,[[34]](#endnote-35) [[35]](#endnote-36) Prognostic Factors,[[36]](#endnote-37) and Scandinavian Stroke Scale[[37]](#endnote-38)). This form may be filled in by medial staff in the admissions unit after appropriate training allowing 24/7 recruitment. It is thus kept as short as possible to avoid the randomisation process interfering with clinical care. Any relevant background details which can be retrieved from the notes will be collected by the researcher at the day 3 assessment.

Patients will be randomised by telephone by simple randomisation. We considered using minimisation to balance prognostic characteristics between the treatment and control groups, but decided against this for the pilot since there is a significant chance of guessing trial allocation with this method with a single centre trial.

We are, however, considering treatment allocation by minimisation using well-established factors predictive of stroke outcome (living alone before the stroke, pre stroke functional independence, normal verbal response, ability to lift the affected arm against gravity and ability to walk unaided)[[38]](#endnote-39) and a measure of hypoxia (block 1: baseline saturation ≤92%, block 2: baseline saturation 93-94%, block 3 baseline saturation ≥95%) for the planned multicentre trial, and have therefore included the minimisation criteria on the randomisation form.

**Follow-up assessments**

This study is designed to be as simple as possible for the recruiting doctor, to allow patient recruitment to be incorporated into the admission process. All follow-up assessments will be done by a member of the research team.

**Over night pulse oximetry and day 3 assessment**

In order to ascertain whether the prescribed oxygen supplementation effectively prevents hypoxia pulse oximetry will be performed over night on night two while the patient is receiving oxygen or control treatment. We will also be able to quantify the time the patients spend with an oxygen saturation above the normal range. This is necessary to fine tune the dosage schedule for the planned outcome study.

The day 3 assessment (Assessment Form 1) will document compliance with the trial treatment, relevant aspects of the patient's past medical history, and the stroke type (Oxfordshire Community Project Stroke Classification), and confirm completion of the over night pulse oximetry. If incomplete, pulse oximetry will be repeated on night 3 of treatment.

**Week 1 assessment**

The week 1 assessment (Assessment form 2) is designed to screen for potential adverse effects of oxygen and includes indicators of infection, stress (tachycardia and hypertension), behaviour disturbance, cognitive function, and neurological deterioration. If the patient is well enough to cooperate the researcher will discuss the 6 month assessment and find out who the preferred contact (s) for mailing of the questionnaire.

**Month 3 assessment**

The month 3 assessment (Assessment Form 3) documents the length of stay in hospital, and discharge destination. The discharge address and the contact details of the GP are recorded in preparation for the 6 month follow-up. It will be completed at 3 months, or before where appropriate. It will be completed using information from the HISS/CHIPS system and patient notes.

**Month 6 assessment**

This assessment (Assessment Form 4) will provide the main outcome (death and disability at 6 months) data for testing the trial hypothesis of the planned larger study. It consists of a questionnaire which will be sent to the patient's preferred contact address (if known) or the discharge address. Outcomes will be assessed using well validated scales (Rankin[[39]](#endnote-40) [[40]](#endnote-41), 3-point Barthel,[[41]](#endnote-42) Euroquol,[[42]](#endnote-43) [[43]](#endnote-44) The Nottingham Extended Activities of Daily Living Index[[44]](#endnote-45) [[45]](#endnote-46)). As in other large studies looking at outcome after acute stroke (e.g. the IST-3 study of thrombolysis for acute stroke currently in progress at out hospital), we will first ascertain whether that patient is still alive (via the hospital computer system and/ or the GP) and then contact the patient by post or by telephone.

This pilot trial aims to assess the likelihood of patients or carers completing the questionnaire. If the relevant information cannot be collected via mail or by phone we will consult patient records and the GP to ascertain outcome data. If a significant proportion of questionnaires is not returned or incomplete we will redesign the questionnaire for the main study or consider alternative methods of ascertaining the main outcomes.

For this pilot we will also collect information on length of stay, readmissions and discharge destination. These data will be gathered using the hospital HISS/CHIPS system (Assessment Form 5). These will allow us to provide basic health economic data. The date and cause of death will be recorded on Assessment Form 6 and contact details for follow up on Assessment Form 7.

**Research Staff**

There will be a full time PhD student (self funding) and a part time research nurse (funded by Geriatric Medicine Research Funds) working on the project.

To allow for 24 hour inclusion of patients by A&E or MAU staff the randomisation procedures are kept very simple. The Trust admits about 2-3 stroke patients per day, and most of those would be randomised by the research team. A&E and MAU staff will only be asked to randomise patients if the unit managers agree with the trial, and if the staff involved are not too busy. While it is expected that the majority of patients will be recruited by the research team, it is considered important for this pilot study, since recruitment by other staff than the core research team will be required for the larger multicentre trial, and any problems with this are likely to become obvious in the pilot phase.

**Main outcomes**

Comparisons will be made between the treatment and control group to determine:

1. Effect size:

- Death and disability (Rankin >3) at 6 months
- Scandinavian Stroke Scale week 1
- Mortality up to 6 months
- Rankin Score at 6 months
- Nottingham EADL score at 6 months

2. Potential Side effects of the study treatment

- Rate of infections (pyrexia or antibiotic treatment at week 1)
- Agitation (sedative or antipsychotic medication at week 1)
- Physiological indicators of stress (tachycardia, hypertension)

3. To assess the feasibility of the study protocol we will examine

- Protocol violations
- Recruitment rate
- The relation between the number of recruited and excluded patients
- The percentage of completed 6-month questionnaires

**DATA ANALYSIS**

**Statistical analysis**

The main comparisons will be made between the control group and the treatment group.

The main analysis will be intention to treat, but a subgroup analysis excluding protocol violators will also be performed. Appropriate parametric and non parametric tests will be used to compare the groups at baseline and after treatment.

**Study size**

This is a pilot study and thus no formal study size calculation was undertaken since no estimate of effect size is available and the study is to be used to determine this as well as the feasibility of the planned multicentre study.

We plan to recruit 150 patients in each of the 2 trial arms. This would enable in the full sample detection of a small to medium effect size (0.4 SD of the main outcomes (Rankin, Nottingham EADL and EuroQuol) with 90% power and with a 5% two sided significance level. The study size was determined considering the following factors:

Ronning et al included almost 600 patients in their study of routine oxygen supplementation and did not find an overall difference on functional outcome at one year. There was a trend towards a better result with oxygen treatment in the 240 subjects with severe stroke, although this finding was not backed up by the use of a suitable test for heterogeneity. Our protocol may be more likely to show benefits because oxygen is given for longer, and titrated according to baseline oxygen saturation.[[46]](#endnote-47) However, given the data of the above study it is likely that any observed effect is small. This pilot study will provide us with data to inform a study size calculation.

Over the last year we performed a small study of the effects of oxygen supplementation for 24 hours within 3 days of admission on oxygen saturation. It showed a significant improvement of oxygen saturation in the treated group, but the study was not powered to look at functional outcomes. It took 6 months to recruit 50 patients for this study.[[47]](#endnote-48) It is therefore likely that we will be able to recruit 300 patients over three years.

**Statistical Advice**

The statistical aspects of study design and analysis have been discussed with Professor Peter Jones and Dr Robert Hills who both have wide experience in statistics of medical trials and trial design, and who are co-applicants. Because we have no data on the expected effect size form other trials or on the SD of the main outcome (death and disability) in our patient population we are not yet in a position to do a study size calculation.

Prof Peter Jones

Department of Mathematics

Keele University

[p.w.jones@maths.keele.ac.uk](mailto:p.w.jones@maths.keele.ac.uk)

**Data Analysis**

Data entry and analysis will be performed by members of the study team (K. Ali, S. Sills, C. Roffe) with advice from P. Jones.

**STUDY TIMETABLE**

July 2004 start of recruitment

July 2005 100 patients recruited

July 2006 200 patients recruited

July 2007 recruitment complete

Jan 2008 follow-up complete

April 2008 statistical analysis complete

October 2008 research paper submitted for publication

**Stroke Oxygen Study**

**Randomisation Form**

Name of person completing this form Centre number

STEP 1 ELIGIBILITY FOR TRIAL INCLUSION

| Admitted with acute stroke within the preceding 24 hours | YES | NO |
| --- | --- | --- |
| Not expected to die within a few months from a non-stroke related illness | YES | NO |
| No definite indication for continuous oxygen treatment | YES | NO |
| No definite contraindication to continuous oxygen treatment | YES | NO |

Please proceed to patient details if all answers to Q1 are YES

**STEP 2 PATIENT DETAILS**

Name Sex Date of birth

Date and time of stroke onset (24 h clock)

Side of hemiparesis

| Right | Left | Neither | Both Sides |
| --- | --- | --- | --- |

Glasgow Coma Scale (please circle one response in each row)

| Eye  Opening | None (1) | To pain  (2) | To speech  (3) | Spontaneous (4) |  |  |
| --- | --- | --- | --- | --- | --- | --- |
| Motor Response | None (1) | Extension  (2) | Abnormal  flexion (3) | Withdrawal (4) | Localizes  to pain (5) | Obeys  commands (6) |
| Verbal response | None (1) | Incomprehensible (2) | Inappropriate (3) | Confused (4) | Oriented  (5) |  |

**STEP 3 PROGNOSTIC FACTORS (please circle the yes or no and complete oxygen saturation)**

| 1. Living alone before the stroke | YES | NO |
| --- | --- | --- |
| 2. Independent in Activities of daily living before the stroke | YES | NO |
| 3. Normal verbal response to questions | YES | NO |
| 4. Able to lift the affected arm against gravity | YES | NO |
| 5. Able to walk unaided | YES | NO |
| 6. Oxygen saturation at randomisation | % | |

STEP 4 CONSENT

| 1. Fully informed consent  Before randomisation either 1  or 2 and 3 must be answered as **YES** | YES | NO |
| --- | --- | --- |
| 2. Patient does not disagree with trial | YES | NO |
| 3. Assent from next of kin | YES | NO |

**STEP 5 RANDOMISATION** Please phone (xxxxx) xxxxxxxx for 24 hour randomisation or any queries

Date and time of randomisation (24 h clock)

Randomisation number

Treatment allocated (circle the treatment the patient was randomised to receive)

| No routine oxygen | 2l/min continuously for 72 hours | 3l/min continuously for 72 hours |
| --- | --- | --- |
|  | 2l/min for 3 nights | 3l/min for 3 nights |

Treatment start time (24 h clock) PTO for Baseline Neurological Assessment

**Stroke Oxygen Study**

**Baseline Neurological Assessment (Admission)**

Name Randomisation no

**Scandinavian Stroke Scale**

**(tick one box in each group, for items marked with* score the affected side)**

**Consciousness:**

- **6** fully conscious
- **4** somnolent,

can be awaked to full consciousness

- **2** reacts to verbal command,

but is not fully conscious

**Orientation:**

- **6** correct for time, place and person
- **4** two of these
- **2** one of these
- **0** completely disorientated

**Speech:**

- **10** no aphasia
- **6** limited vocabulary or incoherent speech
- **3** more than yes/no, but not longer sentences
- **0** only yes/no or less

**Facial palsy:**

- **2** none/dubious
- **0** present

**Eye movement:**

- **4** no gaze palsy
- **2** gaze palsy present
- **0** conjugate eye deviation

**Total Score**

**Arm, motor power *:**

- **6** raises arm with normal strength
- **5** raises arm with reduced strength
- **4** raises arm with flexion in elbow
- **2** can move, but not against gravity
- **0** paralysis

**Hand, motor power *:**

- **6** normal strength
- **4** reduced strength in full range
- **2** some movement, fingertips do not reach palm
- **0** paralysis

**Leg, motor power *:**

- **6** normal strength
- **4** raises straight leg with reduced strength
- **5** raises leg with flexion of knee
- **2** an move, but not against gravity
- **0** paralysis

**Gait:**

- **12** walks 5 m without aids
- **9** walks with aids
- **6** walks with help of another person
- **3** sits without support
- **0** bedridden/wheelchair

**Stroke Oxygen Study**

# Day 3 Assessment (Assessment form 1)

Name Randomisation no

Pulse oximetry night 2 21:00-9:00 complete Yes No

If pulse oximnetry is not complete, repeat on night 3

Compliance with treatment regimen (tick the statement that applies on day 3)

| Oxygen currently in place (2) | Oxygen not in place, but patient and staff report that the patient is receiving oxygen (1) | No evidence of oxygen supplementation  (0) |
| --- | --- | --- |

Medical History (circle yes or no as appropriate)

| COAD or Asthma [by Hx or from list of drugs] | YES | NO |
| --- | --- | --- |
| Other chronic lung problem [kyphoscoliosis, thoracoplasty, pneumoconiosis etc) | YES | NO |
| Heart failure [by Hx or >20 mg frusemide or equivalent per day] | YES | NO |
| Ischaemic heart disease [H/O angina or MI or tx with nitrates or nicorandil] | YES | NO |
| Atrial fibrillation | YES | NO |

Stroke classification (circle the class that reflects the patient's worst symptoms)

| TAC | PAC | LAC | POC | TIA | Unclassifiable |
| --- | --- | --- | --- | --- | --- |

| Date of pulse oximetry |  |
| --- | --- |
| Baseline wake oxygen saturation |  |
| Mean nocturnal oxygen saturation |  |
| The 4% Oxygen Desaturation Index |  |
| The lowest nocturnal oxygen saturation |  |
| The time spent with an oxygen saturation <95% |  |
| The time spent with an oxygen saturation <92% |  |
| The time spent with an oxygen saturation <90% |  |
| The time spent with an oxygen saturation <80% |  |
| The time spent with an oxygen saturation >98% |  |
| The mean nocturnal heart rate |  |

**Stroke Oxygen Study**

**Week 1 (Assessment form 2 a)**

Name Randomisation no

Date of death (if applicable)

Please complete the Notification of Death Form if the patient is deceased.

| Antibiotics prescribed since admission | YES | NO |
| --- | --- | --- |
| Continuous oxygen prescribed outside protocol for clinical indications | YES | NO |
| Sedatives or antipsychotic drugs prescribed since admission | YES | NO |
| Highest temperature during week 1 |  |  |
| **The highest heart rate during the 3 days of trial treatment** |  |  |
| **The highest systolic blood pressure during the 3 days of trial treatment** |  |  |
| **The highest diastolic blood pressure during the 3 days of trial treatment** |  |  |
| Highest temperature during week 1 |  |  |

**CT /MRI diagnosis (please tick one of the boxes)**

- **Cerebral infarct**
- **Primary intracerebral haemorrhage**
- **Subdural haemorrhage**
- **Subarachnoid haemorrhage**
- **Brain tumour**
- **Head scan not performed**
- **Other (please specify) _______________________________**

**Final diagnosis**

**(Please make a final diagnosis using the clinical presentation, time course, head scan. Tick only one of the boxes)**

- **Ischaemic stroke**
- **TIA**
- **Primary intracerebral haemorrhage**
- **Cerebrovascular accident without CT confirmation of aetiology**
- **Other (Please specify) _____________________________**

For patients who were incompetent to sign consent at recruitment:

Competent to sign today? Yes No

If yes, explain study again and ask patient to sign form.

**Stroke Oxygen Study**

**Week 1 (Assessment form 2 b)**

Name Randomisation no

**Scandinavian Stroke Scale**

**(tick one box in each group, for items marked with* score the affected side)**

**Consciousness:**

- **6** fully conscious
- **4** somnolent,

can be awaked to full consciousness

- **2** reacts to verbal command,

but is not fully conscious

**Orientation:**

- **6** correct for time, place and person
- **4** two of these
- **2** one of these
- **0** completely disorientated

**Speech:**

- **10** no aphasia
- **6** limited vocabulary or incoherent speech
- **3** more than yes/no, but not longer sentences
- **0** only yes/no or less

**Facial palsy:**

- **2** none/dubious
- **0** present

**Eye movement:**

- **4** no gaze palsy
- **2** gaze palsy present
- **0** conjugate eye deviation

**Total Score**

**Arm, motor power *:**

- **6** raises arm with normal strength
- **5** raises arm with reduced strength
- **4** raises arm with flexion in elbow
- **2** can move, but not against gravity
- **0** paralysis

**Hand, motor power *:**

- **6** normal strength
- **4** reduced strength in full range
- **2** some movement, fingertips do not reach palm
- **0** paralysis

**Leg, motor power *:**

- **6** normal strength
- **4** raises straight leg with reduced strength
- **5** raises leg with flexion of knee
- **2** an move, but not against gravity
- **0** paralysis

**Gait:**

- **12** walks 5 m without aids
- **9** walks with aids
- **6** walks with help of another person
- **3** sits without support
- **0** bedridden/wheelchair

**Mini Mental State Examination** (see separate form) Total score

For patients who were incompetent to sign consent at recruitment:

Competent to sign today? Yes No

If yes, explain study again and ask patient to sign form.

**Preferred contact for the 6 month follow-up** (please record on the patient contact form)

**Stroke Oxygen Study**

**3 Months follow-up Questionnaire (Assessment form 3)**

Name Randomisation number:

Date of discharge from hospital

Date of death (if applicable)

Please complete the Notification of Death Form if the patient is deceased.

**Discharge destination**

- Own home or home of a relative or friend
- Residential home or Nursing Home
- Continuing care hospital
- Still in hospital

Discharge address:

_____________________________

_____________________________

_____________________________

GP:

_____________________________

_____________________________

_____________________________

**Stroke Oxygen Study**

**6 Months follow-up Questionnaire (Assessment from 4)**

**Name:**

**Date of birth:**

**Where do you live now?** Please tick the box which applies to you

- Im my own home
- In the home of a relative
- In a residential home
- In a nursing home
- In a continuing care hospital
- I have not yet left hospital after my stroke

**Who do you live with ?** Please tick the box which applies to you

- I live alone
- I live with a spouse/partner
- I live with family/friends

**Have you been admitted to hospital again for any reason after you were discharged?**

- Yes (once)
- Yes (more than once)
- No

**Are you left with any symptoms or problems after your stroke?** Please tick the tick one box next to the statement which best describes your present state

- I have no symptoms at all.
- I have a few symptoms, but these do not interfere with my everyday life.
- I am unable to carry out all previous activities, but am able to look after my own affairs without assistance.
- I need some help with looking after my own affairs, but am able to walk without assistance.
- I am unable to walk without assistance and unable to attend to my own bodily needs without assistance, but I do not need constant care and attention.
- I have major symptoms which severely handicapped me and I need constant attention day and night.

**This page contains some more specific questions on how the stroke has affected your day to day life and physical functioning**

**Bladder control**

Please tick the box next to the statement which best describes your bladder control

- urinary catheter
- incontinent
- occasional accidents (once a week)
- continent

**Transfers from bed to chair**

Please tick the box next to the statement which best describes your ability to get from bed into a chair

- unable
- able with major help (one or two people)
- able with minor help (verbal or physical)
- independent

**Mobility**

Please tick the box next to the statement which best describes your mobility

- Immobile
- Wheelchair independent
- Walking with the help of one person (verbal or physical)
- independent

**Memory**

Please tick the box next to the statement which best describes your memory

- My memory is as good as before the stroke
- My memory has deteriorated since the stroke

**These questions will help us to find whether your wellbeing is affected by any of your medical problems. Some questions may address similar topics than the previous pages, but this will help us to get more specific information about how well you are**

**Mobility**

Please tick the box which best describes your level of mobility

- I have no problems walking
- I have some problems walking
- I am confined to bed

**Self care**

Please tick the box which best describes your level of mobility

- I have no problems with self care
- I have some problems washing and dressing
- I am unable to wash or dress myself

**Pain or discomfort**

Please tick the box next to the statement which best describes your level of pain or discomfort

- I have no pain or discomfort
- I have moderate pain or discomfort
- I have extreme pain or discomfort

**Anxiety and depression**

Please tick the box next to the statement which best your level of anxiety and depression

- I am not anxious or depressed
- I am moderately anxious or depressed
- I am extremely anxious or depressed

**The EuroQuol Questionnaire**

To help you to say how good (or bad) your health is we have drawn a scale (rather like a thermometer) on which the best health you can imagine is marked by 100 and the worst health you can imagine is marked by 0.

We would like you to indicate on this scale how good or bad your health is in your own opinion today.

Please do this by drawing a line from the box below to whichever point on the scale indicates how good or bad your current health is.

Your own health today is:

0

Worst imaginable health state

Best imaginable health state

100

**This is a long list of questions designed to find out if and how your medical condition has affected your ability to do day to day activities and to pursue work and leisure interests**

**Do you** [please tick the option that describes your activities best]

|  | **Alone easily** | **Alone with difficulty** | **With help** | **Not at all** |
| --- | --- | --- | --- | --- |
| **Mobility:** |  |  |  |  |
| Walk around inside? |  |  |  |  |
| Climb stairs? |  |  |  |  |
| Get in and out of the car? |  |  |  |  |
| Walk on uneven ground? |  |  |  |  |
| Travel on public transport? |  |  |  |  |
| Drive a car? |  |  |  |  |
| **Eating and Drinking:** |  |  |  |  |
| Manage to feed yourself? |  |  |  |  |
| Manage to make a hot drink? |  |  |  |  |
| Take hot drinks from one room to another? |  |  |  |  |
| Do the washing up? |  |  |  |  |
| Make yourself a hot snack? |  |  |  |  |
| **Money and Shopping:** |  |  |  |  |
| Manage your own Money when out? |  |  |  |  |
| Do your own shopping? |  |  |  |  |
| **Housework:** |  |  |  |  |
| Wash small items of clothing? |  |  |  |  |
| Do a full clothes wash? |  |  |  |  |
| **Leisure and communication:** |  |  |  |  |
| Read newspapers and books? |  |  |  |  |
| Use the telephone? |  |  |  |  |
| Write letters? |  |  |  |  |
| Go out socially? |  |  |  |  |
| Manage your own garden? |  |  |  |  |

**This is the last page of the questionnaire**

**Can you remember which of the treatment you were given as part of this trial?**

- Oxygen for 3 days
- Oxygen only if needed
- I don't know

**I completed the form** [tick what applies to you]

- On my own
- With some help from a relative, friend or carer
- A relative, friend or carer completed the form for me

Thank you very much for helping with this research.

**Stroke Oxygen Study**

**6- Months Discharge Form (Assessment form 5)**

Name Randomisation number:

Date of discharge from hospital

Date of death (if applicable)

Please complete the Notification of Death Form if the patient is deceased.

**Discharge destination**

- Own home or home of a relative or friend
- Residential home or Nursing Home
- Continuing care hospital
- Still in hospital

**Readmissions** (include MAU and A&E presentations with discharge home)

Admission date _____________________________

Discharge Date_____________________________

Hospital _____________________________

Main diagnosis_____________________________

Admission date _____________________________

Discharge Date_____________________________

Hospital _____________________________

Main diagnosis_____________________________

Admission date _____________________________

Discharge Date_____________________________

Hospital _____________________________

Main diagnosis_____________________________

**Stroke Oxygen Study**

**Notification of Death (Assessment Form 6)**

Name Randomisation number:

**Date of Death**

**Has the cause of death been confirmed by autopsy?** Yes No

**Likely cause of death** (tick one box only)

- Neurological damage due to the initial stroke
- Recurrent stroke
- Pneumonia
- Other infection
- Pulmonary Embolism
- Ischaemic heart disease
- Other cause of death (please specify) _______________________________

**Stroke Oxygen Study**

**Patient contact details (Assessment form 7)**

Name Randomisation number:

**Home address**

Road _______________________________________

Town _______________________________________

Post Code _______________________________________

Telephone _______________________________________

**Discharge address**

Road _______________________________________

Town _______________________________________

Post Code _______________________________________

Telephone _______________________________________

**Consultant looking after the patient during the hospital episode**

Name _______________________________________

Address _______________________________________

Telephone _______________________________________

Fax _______________________________________

Secretary _______________________________________

Telephone _______________________________________

Fax _______________________________________

**Details of a reliable contact**

Name _______________________________________

Relationship to patient ________________________________

Road _______________________________________

Town _______________________________________

Post Code _______________________________________

Telephone _______________________________________

**General Practitioner**

Name _______________________________________

Road _______________________________________

Town _______________________________________

Post Code _______________________________________

Telephone _______________________________________

**References**

1. Stroke Unit Trialists' Collaboration. Organised inpatient (stroke unit) care for stroke (Cochrane Review). In: The Cochrane Library, Issue 4, 2003. Chichester, UK: John Wiley & Sons, Ltd. [↑](#endnote-ref-2)
2. Indredavik B, Bakke F, Slordahl SA, Rosketh A, Haheim LL. Treatment in a combined acute and rehabilitation stroke unit. Stroke 1999;30:917-23. [↑](#endnote-ref-3)
3. Roffe C. Hypoxaemia and stroke. Reviews in Clinical Gerontology 2001;11:323-335. [↑](#endnote-ref-4)
4. Lewis LD, Ponten U, Siesjo BK. Homeostatic regulation of brain energy metabolism in hypoxia. Acta Physiol Scand. 1973 Jun;88(2):284-6. [↑](#endnote-ref-5)
5. Nakajima S, Meyer JS, Amano T, Shaw T, Okabe T, Mortel KF. Cerebral vasomotor responsiveness during 100% oxygen inhalation in cerebral ischemia. Arch Neurol. 1983;40(5):271-6. [↑](#endnote-ref-6)
6. Yager JY, Thornhill JA. the effect of age on susceptibility to hypoxic-ischaemic brain damage. Neuroscience and Biobehavioural Reviews 1997;21(2):167-174. [↑](#endnote-ref-7)
7. Yamamoto M, Meyer JS, Sakai F, Yamaguchi F. Aging and cerebral vasodilator responses to hypercarbia: responses in normal aging and in persons with risk factors for stroke. Arch Neurol. 1980;37:489-96. [↑](#endnote-ref-8)
8. Arora A, Gray R, Crome P, Roffe C. British Association of Stroke Physicians (BASP) members' views on oxygen treatment in acute stroke. Age Ageing 2003;32(suppl 2):41 [abstract]. [↑](#endnote-ref-9)
9. Ogburn-Russell L, Johnson JE. Oxygen saturation levels in the well elderly: altitude makes a difference. J Gerontol Nurs 1990;16:26-30. [↑](#endnote-ref-10)
10. Leach RM, Bateman NT. Acute oxygen therapy. Br J Hosp Med 1993;49:637-643. [↑](#endnote-ref-11)
11. McCord JM.Oxygen-derived free radicals in postischemic tissue injury.N Engl J Med. 1985 Jan 17;312(3):159-63. [↑](#endnote-ref-12)
12. Ronning OM, Guldvog B. Should stroke victims routinely receive supplemental oxygen? A quasi-randomized controlled trial. Stroke 1999;30:2033-2037. [dup] [↑](#endnote-ref-13)
13. Rawles JM, Kenmure AC. Controlled trial of oxygen in uncomplicated myocardial infarction. Br Med J. 1976 ;1:1121-3. [↑](#endnote-ref-14)
14. <http://www.rcplondon.ac.uk/pubs/books/stroke/index.htm> [↑](#endnote-ref-15)
15. Adams HP Jr, Adams RJ, Brott T, del Zoppo GJ, Furlan A, Goldstein LB, Grubb RL, Higashida R, Kidwell C, Kwiatkowski TG, Marler JR, Hademenos GJ; Stroke Council of the American Stroke Association.Guidelines for the early management of patients with ischemic stroke: A scientific statement from the Stroke Council of the American Stroke Association. Stroke. 2003;34:1056-83. [↑](#endnote-ref-16)
16. European Stroke Initiative Executive Committee and the EUSI Writing Committee. European Stroke Initiative Recommendations for Stroke Management - Update 2003. Cerebrovasc Dis 2003;16:311-337. http://www.eusi-stroke.com/recommendations/rc_overview.shtm [↑](#endnote-ref-17)
17. Arora A, Gray R, Crome P, Roffe C. British Association of Stroke Physicians (BASP) members' views on oxygen treatment in acute stroke. Age Ageing 2003;32(suppl 2):41 [abstract]. [dup] [↑](#endnote-ref-18)
18. Ronning OM, Guldvog B. Should stroke victims routinely receive supplemental oxygen? A quasi-randomized controlled trial. Stroke 1999;30:2033-2037. [dup] [↑](#endnote-ref-19)
19. European Stroke Initiative Executive Committee and the EUSI Writing Committee. European Stroke Initiative Recommendations for Stroke Management - Update 2003. Cerebrovasc Dis 2003;16:311-337. <http://www.eusi-stroke.com/recommendations/rc_overview.shtm> [dup] [↑](#endnote-ref-20)
20. Adams HP Jr, Adams RJ, Brott T, del Zoppo GJ, Furlan A, Goldstein LB, Grubb RL, Higashida R, Kidwell C, Kwiatkowski TG, Marler JR, Hademenos GJ; Stroke Council of the American Stroke Association.Guidelines for the early management of patients with ischemic stroke: A scientific statement from the Stroke Council of the American Stroke Association. Stroke. 2003;34:1056-83. [dup] [↑](#endnote-ref-21)
21. Acid-base balance and blood gases. In: Clinical Laboratory Diagnosis. Ed: Thomas L. TH-Books.1st Ed, Frankfurt 1998. [↑](#endnote-ref-22)
22. Ogburn-Russell L, Johnson JE. Oxygen saturation levels in the well elderly: altitude makes a difference. J Gerontol Nurs 1990;16:26-30. [dup] [↑](#endnote-ref-23)
23. Roffe C, Sills S, Halim M, Wilde K, Allen M, Jones PW, Crome P. Unexpected nocturnal hypoxia in patients with acute stroke. Stroke 2003;34:2641-2645. [↑](#endnote-ref-24)
24. Sills S, Halim M, Roffe C. A pilot study of routine oxygen supplementation in patients with acute stroke.Forthcoming in Age Ageing 2003 [abstract]. [↑](#endnote-ref-25)
25. Personal Communication. Ali K, Sills S, Roffe C. The effect of different doses and routes of oxygen administration on oxygen saturation in stroke patients. Forthcoming in Age Ageing 2004. [↑](#endnote-ref-26)
26. Arora A, Gray R, Crome P, Roffe C. British Association of Stroke Physicians (BASP) members' views on oxygen treatment in acute stroke. Age Ageing 2003;32(suppl 2):41 [abstract]. [dup] [↑](#endnote-ref-27)
27. Sills S. Nocturnal oxygen saturation during the first three nights after hospital admission for acute stroke. Mphil thesis, submitted to Keele University in February 2004. [↑](#endnote-ref-28)
28. Baron J. Mapping the ischaemic penumbra with PET: implications for acute stroke treatment. Cerebrovascular Diseases 1999; 9:193-201. [↑](#endnote-ref-29)
29. Ronning OM, Guldvog B. Should stroke victims routinely receive supplemental oxygen? A quasi-randomized controlled trial. Stroke 1999;30:2033-2037. [dup] [↑](#endnote-ref-30)
30. World Health Organization. Cerebrovascular Disorders: A Clinical and Research Classification. Geneva, Switzerland: World Health Organization; 1998:43. [↑](#endnote-ref-31)
31. . European Carotid Surgery Trialists' Collaborative Group. MRC European Carotid Surgery Trial: interim results for symptomatic patients with severe (70-99%) or with mild (0-29%) carotid stenosisLancet 1991 May 25;337(8752):1235-43. [↑](#endnote-ref-32)
32. [No authors listed]. Randomised trial of endarterectomy for recently symptomatic carotid stenosis: final results of the MRC European Carotid Surgery Trial (ECST)
    Lancet. 1998 May 9;351(9113):1379-87. [↑](#endnote-ref-33)
33. Http://www.dcn.ed.ac.uk/ist3 [↑](#endnote-ref-34)
34. Teasdale G, Jennett B. Assessment of coma and imparied consciousness. A practical scale. Lancet 1974;2:81-8. [↑](#endnote-ref-35)
35. Levy DE, Bates D, Caronna JJ, Cartlidge NE, Knill-Jones RP, Lapinski RH, Singer BH, Shaw DA, Plum F. Prognosis in nontraumatic coma. Ann Intern Med. 1981 Mar;94(3):293-301. [↑](#endnote-ref-36)
36. Weir NU, Counsell CE, McDowall M, Gunkel A, Dennis SM. Reliablility of the variables in a new set of models that predict outcome after stroke. J Neurosurg Psychiatry 2002;74:447-45. [↑](#endnote-ref-37)
37. Multicenter trial of hemodilution in ischemic stroke. Background and study protocol. Scandinavian Stroke Study Group. Stroke 1985;16:885-90. [↑](#endnote-ref-38)
38. Weir NU, Counsell CE, McDowall M, Gunkel A, Dennis SM. Reliablility of the variables in a new set of models that predict outcome after stroke. J Neurosurg Psychiatry 2002;74:447-45. [↑](#endnote-ref-39)
39. Rankin J. Cerebral vascular accidents in patients over the age of 60. 2. Prognosis. Scottish Medical Journal 1957;2:200-215. [↑](#endnote-ref-40)
40. Sulter G, Steen C, De Keyser J. Use of the Barthel index and modified Rankin scale in acute stroke trials.

    Stroke. 1999 Aug;30(8):1538-41. [↑](#endnote-ref-41)
41. Ellul J, Watkins C, Barer D. Estimating the total Barthel Scores from just three items: The European Stroke Database minimum dataset for assessing functional status at discharge from hospital. Age and Ageing 1998;27:115-122. [↑](#endnote-ref-42)
42. Dorman PJ, Waddell FM, Slattery J, Dennis MS, Sandercock PAG. Is the EuroQol a valid measure of health-related quality of life after stroke? Stroke. 1997;28:1876–1882. [↑](#endnote-ref-43)
43. Dorman P, Slattery J, Farrell B, Dennis M, Sandercock P. Qualitative comparison of the reliability of health status assessments with the EuroQol and SF-36 questionnaires after stroke. United Kingdom Collaborators in the International Stroke Trial. Stroke. 1998 Jan;29(1):63-8. [↑](#endnote-ref-44)
44. Nouri FM, Lincoln NB. An extended Activities of daily living scale for stroke patietns. Clinical Rehabilitaiton 1987;1:301-305 [↑](#endnote-ref-45)
45. Duncan PW, Jorgensen HS, Wade DT. Outcome measures in acute stroke trials. A systematic review and some recommendations to improve practice. Stroke 2000; 31:1429-1438. [↑](#endnote-ref-46)
46. Ronning OM, Guldvog B. Should stroke victims routinely receive supplemental oxygen? A quasi-randomized controlled trial. Stroke 1999;30:2033-2037. [dup] [↑](#endnote-ref-47)
47. Personal Communication, S. Sills, C. Roffe 2003. [↑](#endnote-ref-48)
